# Impact of Counseling on the Hospital Cornea Retrieval Programme: A Prospective Study

**DOI:** 10.7759/cureus.86402

**Published:** 2025-06-19

**Authors:** Anjna S Thakur, Aditi Dubey, Kavita Kumar

**Affiliations:** 1 Department of Ophtalmology, Gandhi Medical College, Bhopal, IND

**Keywords:** awareness, corneal blindness, counseling, eye banking, eye donation, grief counselor, hospital cornea retrieval programme, willingness

## Abstract

Background

Corneal blindness is a major public health concern worldwide and poses a significant burden in India. It can severely impact an individual’s quality of life, particularly when access to timely and effective treatment is limited. One of the key strategies to combat this issue is the Hospital Cornea Retrieval Programme (HCRP), which aims to increase the availability of corneal tissue for transplantation. The effectiveness of this program, however, relies heavily on successful counseling efforts to encourage eye donation and support the retrieval process.

Aim and objective

The main objective of this study is to assess the impact of counseling on awareness and willingness for eye donation within the HCRP.

Methods

This 18-month, prospective, hospital-based observational study was conducted at Gandhi Medical College and the associated Hamidia Hospital in Bhopal, India. The study included 58 attendants of critically ill and deceased patients. Trained grief counselors established rapport with families, offering assistance with medical and legal formalities while gradually educating them about eye donation. Participant awareness and willingness were assessed using a structured questionnaire before and after counseling.

Results

Before counseling, 53.4% of participants were entirely unaware of cornea retrieval, 44.8% had partial awareness, and only 1.7% were fully informed. Post-counseling, no participants remained completely unaware, with 74.1% achieving partial awareness and 25.9% becoming fully aware. Initially, none of the participants were willing to donate eyes, but after counseling, 10.3% expressed willingness. Education level and socioeconomic status were significant factors influencing willingness, with 27.8% of graduates and 30% of participants from the upper socioeconomic class willing to donate after counseling.

Conclusion

Counseling significantly improved awareness across all demographic groups but had a more modest effect on willingness to donate. These findings highlight the need for targeted, culturally sensitive approaches that address not only knowledge gaps, but also deeper cultural and personal concerns to increase corneal donation rates in India.

## Introduction

Corneal blindness represents a significant global health challenge, affecting approximately six million people worldwide as the fifth leading cause of blindness [1]. In India, the situation is particularly concerning, with an estimated prevalence of 0.45% in the general population - translating to approximately 6.8 million individuals with unilateral corneal blindness and one million with bilateral corneal blindness [2,3]. This substantial burden not only impacts individuals' quality of life, but also places considerable socioeconomic strain on the healthcare system and society at large.

While the majority of corneal blindness cases are preventable or treatable, a significant gap persists between the demand for corneal tissue and its availability [4]. Corneal transplantation remains the primary treatment modality for corneal blindness, but the shortage of donor corneas presents a major limitation to addressing this public health issue effectively. This disparity underscores the urgent need to increase corneal donation rates and improve the efficiency of eye banking systems throughout the country [5]. While India procures a substantial number of donor corneas annually, the corneal utilization rate remains significantly lower - estimated at around 50% - highlighting the urgent need to optimize donor conversion and improve tissue quality and processing standards [6].

The Hospital Cornea Retrieval Programme (HCRP), launched in 1991 under the direction of the Eye Bank Association of India (EBAI), represents a crucial initiative aimed at addressing this challenge [7]. This program focuses on retrieving corneal tissues from eligible and willing donors after death in hospital settings and has become a major source of corneas for eye banks. The core strategy of HCRP focuses on providing compassionate grief counseling and motivational support to bereaved families in hospital settings, creating opportunities for corneal donation discussions during these sensitive moments [8].

Despite the potential of the HCRP, several factors influence the success of eye donation initiatives. These include lack of awareness, misinformation, religious and cultural beliefs, inadequate infrastructure, and insufficient family support [9,10]. Education level and socioeconomic status also play significant roles in shaping attitudes toward eye donation. In this context, Eye Donation Counselors (EDCs) play a paramount role, providing counseling and motivating family members of the deceased to consent to eye donation [11]. Their effectiveness in increasing awareness and willingness to donate has been demonstrated in various studies, emphasizing the potential impact of targeted counseling interventions [12].

The complexity of the eye donation process is further compounded by the emotional state of bereaved families and the time-sensitive nature of corneal retrieval. Counselors must navigate these challenges while providing accurate information and emotional support [13]. Moreover, the diversity of India's population - in terms of culture, religion, and socioeconomic status - necessitates a nuanced and adaptable approach to counseling.

This study fills an important gap in the literature by prospectively evaluating the real-time impact of trained grief counseling on both awareness and actual willingness to donate eyes within the HCRP framework. Unlike previous studies that have primarily focused on awareness levels or retrospective data, this research examines demographic variations, identifies predictors of willingness, and highlights the persistent disconnect between knowledge and conversion. These findings offer actionable insights to optimize eye donation strategies in India.

The primary objective of this study was to evaluate the change in awareness about eye donation among attendants of critically ill or deceased patients after structured counseling. The secondary objectives included assessing the change in willingness to donate and measuring the actual conversion to eye donation post-counseling. By examining the effects of counseling across diverse demographic groups, the present study seeks to identify key factors influencing donation decisions and potential strategies for enhancing the success of corneal retrieval programs.

## Materials and methods

This was a prospective, hospital-based observational study conducted over a period of 18 months, from August 2022 to June 2024. The study was conducted in the Department of Ophthalmology, Gandhi Medical College, and Hamidia Hospitals, a tertiary care center in central India. The study protocol was approved by the Institutional Ethics Committee of Gandhi Medical College, Bhopal (Ethical Clearance Certificate No. 32245/MC/IEC/2022), and informed written consent was obtained from all participants before inclusion in the study.

This prospective study included all attendants and guardians of deceased and critically ill subjects with healthy corneas admitted in ICUs of various departments, emergency, medical wards, mortuary, and referred patients from other departments of Hamidia Hospital. Relatives of deceased subjects and critically ill patients with healthy corneas who were willing to participate in the study were included. Exclusion criteria encompassed patients in the pediatric age group, patients with corneal scarring or any infectious disease in ocular tissue, patients with contraindications for eye donation, and deceased subjects with unknown etiologies. All patients and attendants who were counseled during the study period under HCRP, fulfilling the inclusion criteria, were included.

Socioeconomic status was classified according to the Modified Kuppuswamy Scale (2022), which considers education, occupation, and monthly family income. Educational status was categorized into three groups: illiterate, up to 12th standard, and graduate or above, based on the highest level of formal education completed.

For analysis, age was stratified using a cut-off at 40 years, a threshold commonly used in health behavior studies to distinguish younger adults from middle-aged and older individuals. This cut-off also aligned with observed attitudinal differences in previous eye donation literature and provided balanced subgroup distribution in our sample.

The study involved trained grief counselors specifically designated for grief counseling under the HCRP, who undertook in-depth visits to departments with high mortality rates. Two trained grief counselors participated in this study. Both had prior experience in grief counseling and were trained under the HCRP protocol. Though they differed slightly in age and professional background, they followed a standardized counseling framework using a structured communication template developed by the institutional eye bank team. This protocol included stepwise rapport building, information delivery, myth addressing, and support with formalities. The use of a common counseling format aimed to ensure consistency across interactions and minimize observer- or counselor-related bias. The counselors established rapport with relatives and families of patients to understand their problems and offer help and advice. This bonding often helped relatives open up to the counselors about challenges they faced. Attendants were informed about the utilization of corneas for optical, therapeutic, and research purposes.

Structured counseling protocol

Counseling followed a structured, stepwise protocol based on HCRP guidelines. The stages included: (1) initial rapport-building and offering emotional and legal support; (2) gradual introduction of the concept of eye donation tailored to the family’s emotional readiness; (3) dispelling myths and addressing religious and cultural concerns using verified information; (4) explaining the benefits and process of corneal donation, including its non-disfiguring nature; and (5) offering the option to donate only after the death of the patient, emphasizing voluntary and respectful decision-making.

Counselors followed up with patients over a period of one to three days and provided all possible assistance to attendants and relatives regarding treatment-related issues, medicolegal formalities, and other help. Counseling was initiated with families of critically ill patients to build rapport and provide medical, legal, and emotional support. Educational information regarding eye donation was shared gradually during this period. However, formal documentation of willingness for eye donation was conducted only after the death of the patient, once bereavement counseling was completed and consent was obtained. No active solicitation or consent for donation was pursued during the critical illness phase to ensure ethical compliance and respect for family circumstances.

During this period, they gradually and empathetically educated families about eye donation and addressed myths related to it. While rapport-building and general support were initiated during the patient’s critical illness phase, formal eye donation counseling and consent were obtained only after the patient’s death. Only those families who were initially contacted prior to the patient’s death and subsequently completed the post-mortem counseling and questionnaire were included in the final analysis.

Upon the death of the patient, families were counseled and offered assistance with medicolegal and other formalities. Willingness to donate the eyes of the deceased was noted. Participants completed a pre-prepared, structured questionnaire. The structured questionnaire was administered in two phases: initially, before the counseling session to capture baseline awareness and willingness, and re-administered after counseling to assess changes in participants’ responses. This pre- and post-counseling evaluation enabled direct assessment of the intervention’s impact. Factors related to willingness for eye donation that influenced conversion to actual donation were evaluated.

Awareness and willingness were assessed using a pre-tested, structured, 13-item questionnaire (see Appendices). Items covered general knowledge of eye donation, corneal tissue use, donation procedures, legal and religious aspects, and myths. Awareness was scored based on the number of correct or affirmative responses: (1) not aware, 0 correct responses; (2) partially aware, 1-6 correct responses; (3) fully aware, 7-13 correct responses.

The 13-item awareness questionnaire assessed knowledge of eye donation benefits, timing, procedures, myths, and religious considerations. Each item was given equal weight (1 point per correct or affirmative response), with a total possible score ranging from 0 to 13. To operationalize awareness levels: a score of 0 (no correct or affirmative responses) was categorized as not aware; scores between 1 and 6 were categorized as partially aware, indicating limited or fragmented knowledge; and scores between 7 and 13 were categorized as fully aware, reflecting a more complete and accurate understanding.

These thresholds were selected based on the midpoint (6.5) of the total possible score range and ensured meaningful stratification of participant awareness levels. For illiterate participants, the questionnaire was verbally administered in Hindi using a standardized script. Efforts were made to ensure that the person administering the questionnaire was not the same as the one providing counseling, to reduce interviewer bias. Responses were recorded by the interviewer during both pre- and post-counseling sessions. While the questionnaire was designed specifically for this study, the items were developed with reference to existing eye donation awareness tools and aligned with themes emphasized in EBAI educational materials. The instrument underwent face validation by ophthalmology and public health faculty and was pilot-tested on five participants to ensure clarity and consistency. However, the tool was not formally validated for construct validity or reliability, which is acknowledged as a limitation.

Willingness was recorded as a direct binary response (yes/no) to the question, “Are you willing to donate the eyes of your relative or pledge your own eyes for donation?”

Blood samples of subjects included in the study were tested for serological markers, including human immunodeficiency virus (HIV), Venereal Disease Research Laboratory (VDRL), hepatitis B surface antigen (HBsAg), and antibody to hepatitis C virus (Anti-HCV). Gross examination of corneas was performed by a corneal consultant, and corneal grading was done for endothelial count.

Data were collected through interviews using the pre-prepared questionnaire and compiled using MS Excel (Microsoft® Corp., Redmond, WA, USA). For statistical analysis, the Chi-square test was used. A probability value (p-value) less than 0.05 was considered statistically significant.

## Results

The present study comprised 58 attendants and guardians of deceased and critically ill patients. Among these, male participants were more prevalent than female participants, with one individual identifying as transgender. A majority of participants (74.1%) were above 40 years of age, while 25.9% were below 40 years. Regarding religious affiliation, most participants identified as Hindu, followed by Muslims, with smaller numbers of Sikhs, Sindhis, and one Jain participant (Table 1).

**Table 1:** Demographic Characteristics of Study Participants (n = 58) Data are expressed as number of patients (percentage).

Demographic Variable	Category	n (%)
Gender	Male	50 (86.2%)
	Female	7 (12.1%)
	Transgender	1 (1.7%)
Age Group	< 40 Years	15 (25.9%)
	≥ 40 Years	43 (74.1%)
Religion	Hindu	43 (74.1%)
	Muslim	11 (19.0%)
	Jain	1 (1.7%)
	Sikh	1 (1.7%)
	Sindhi	2 (3.4%)
Socioeconomic Status	Upper Class	10 (17.2%)
	Upper Middle Class	20 (34.5%)
	Lower Middle Class	15 (25.9%)
	Upper Lower Class	12 (20.7%)
	Lower Class	1 (1.7%)
Educational Status	Illiterate	3 (5.2%)
	≤ 12th Standard	37 (63.8%)
	Graduate or Above	18 (31.0%)

The socioeconomic classification of participants showed a varied distribution, with 17.2% belonging to the upper class, 34.5% to the upper middle class, 25.9% to the lower middle class, 20.7% to the upper lower class, and 1.7% to the lower class. Educationally, 5.2% of the participants were illiterate, 63.8% had received education up to the 12th standard, and 31.0% were graduates or had higher education (Table 1).

A significant improvement in awareness regarding cornea retrieval was observed following counseling. Initially, 53.4% of participants were entirely unaware of cornea retrieval, 44.8% had partial awareness, and only 1.7% were fully informed. After the counseling session, none of the participants remained completely unaware, while 74.1% attained partial awareness, and 25.9% achieved full awareness (Table 2).

**Table 2:** Awareness Among Participants Before and After Counseling N: Number of participants; %: Percentage

Awareness Level	Before Counseling		After Counseling		Change (%)
	N	%	N	%	
Not Aware (0 Positive Responses)	31	53.4	0	0.0	-53.4
Partially Aware (1-6 Positive Responses)	26	44.8	43	74.1	+29.3
Fully Aware (7-13 Positive Responses)	1	1.7	15	25.9	+24.2
Total	58	100.0	58	100.0	-

In terms of willingness to donate eyes, none of the participants expressed willingness prior to counseling. However, post-counseling, six participants (10.3%) indicated a willingness to donate eyes (Table 3). Gender-wise, this included five male participants (10.0% of males) and one female participant (14.3% of females) (Table 4). When analyzed by age group, one participant (6.7%) from the under-40 group and five participants (11.6%) from the over-40 group expressed willingness to donate post-counseling (Table 4).

**Table 3:** Distribution of Participants Before and After Counselling as per Willingness and Conversion to Eye Donation N: Number of participants; %: Percentage

Awareness Level	Before Counseling		After Counseling		Change (%)
	N	%	N	%	
Unwillingness for Eye Donation	40	69.0	22	38.0	-31.0
Willingness for Eye Donation	18	31.0	30	51.7	+20.7
Actual Conversion for Eye donation	0	0.0	6	10.3	+10.3
Total	58	100.0	58	100.0	-

**Table 4:** Demographic Factors Influencing Awareness and Willingness After Counseling The Chi-square test was used, and *p-value of <0.05 was considered statistically significant. N: Number of participants; %: Percentage

Demographic Factors	Awareness Levels After Counseling		Willingness for Eye Donation (%)	Actual Conversion (%)	Chi-Square Statistic	p-value
	Fully Aware (N, %)	Partially Aware (N, %)				
Gender						
Female	3 (42.9)	4 (57.1)	14.3	14.3	1.494	0.474
Male	12 (24.0)	38 (76.0)	10.0	10.0		
Transgender	0 (0.0)	1 (100.0)	0.0	0.0		
Age Group (Years)						
18-25	5 (62.5)	3 (37.5)	12.5	12.5	7.722	0.048*
25-40	2 (25.0)	6 (75.0)	12.5	0.0		
40-60	7 (21.9)	25 (78.1)	12.5	12.5		
> 60	1 (10.0)	9 (90.0)	10.0	10.0		
Religion						
Hindu	11 (25.6)	32 (74.4)	11.6	11.6	3.926	0.416
Muslim	3 (27.3)	8 (72.7)	0.0	0.0		
Jain	1 (100.0)	0 (0.0)	100.0	100.0		
Sikh	0 (0.0)	1 (100.0)	0.0	0.0		
Sindhi	0 (0.0)	2 (100.0)	0.0	0.0		
Socioeconomic Status						
Upper Class	3 (33.3)	6 (66.7)	30.0	20.0	7.154	0.307
Upper Middle	7 (35.0)	13 (65.0)	5.0	5.0		
Lower Middle	4 (26.7)	11 (73.3)	13.3	6.7		
Upper Lower	0 (0.0)	9 (100.0)	0.0	0.0		
Lower	1 (20.0)	4 (80.0)	0.0	0.0		
Education						
Illiterate	0 (0.0)	3 (100.0)	0.0	0.0	7.729	0.357
≤ Class 12th	7 (18.9)	30 (81.1)	2.7	0.0		
Graduate	8 (44.4)	10 (55.6)	27.8	16.7		

Religion-wise, 5 out of 43 Hindu participants (11.6%) and the single Jain participant (100%) expressed willingness to donate eyes following counseling, whereas none of the Muslim, Sikh, or Sindhi participants were willing (Table 3). Socioeconomic status also appeared to influence willingness, with the upper class showing the highest rate of willingness (30.0%), followed by the lower middle class (13.3%) and the upper middle class (5.0%). Participants from the upper lower and lower classes did not express any willingness to donate (Table 4).

Education level demonstrated a strong association with willingness to donate. None of the illiterate participants expressed willingness, while 2.7% of those educated up to 12th class, and 27.8% of those with graduate-level education or higher, indicated a willingness to donate eyes (Table 4).

## Discussion

The findings of this study clearly demonstrate that structured counseling significantly improves awareness regarding cornea retrieval. The increase from 53.4% complete unawareness to 100% awareness (either partial or full) post-counseling illustrates the power of direct and personalized health communication, supporting the observations of Sharma et al., who emphasized that knowledge gaps about eye donation persist, despite existing awareness campaigns [5].

Despite the significant rise in awareness, the actual willingness to donate eyes remained relatively low, with only 10.3% of participants agreeing to eye donation after counseling. The 10.3% conversion rate observed in this study, while modest, is comparable to rates reported in early-phase Indian HCRP studies, which typically range between 5% and 15% in non-mandatory counseling settings. For example, studies by Aggarwal et al. [8] and Sharma et al. [9] reported conversion rates of 8%-12% in tertiary hospitals with active counseling.

Although India lacks an official benchmark for ideal conversion under HCRP, the EBAI and WHO recommend significantly higher rates for program sustainability - typically above 30%-40% in mature eye banking systems. Thus, while our findings indicate meaningful impact, they also underscore the need for systemic, cultural, and procedural enhancements to achieve optimal conversion levels.

This aligns with findings by Aggarwal et al., who highlighted that obtaining consent remains a significant challenge, even with targeted educational interventions [8]. These results suggest that, while awareness is necessary, it alone may not be sufficient to influence action.

A gender-wise analysis revealed a slight difference in willingness, with 14.3% of females and 10.0% of males showing readiness to donate, though the difference was not statistically significant. This observation corroborates the study by Acharya et al., which reported that gender does not play a significant role in influencing decisions regarding eye donation [11].

Interestingly, a higher percentage of participants over 40 years were willing to donate eyes compared to their younger counterparts (11.6% vs. 6.7%). This finding contrasts with Sharma et al., who observed higher willingness among younger individuals [9]. Such variation implies that counseling strategies might need to be tailored based on age demographics to achieve better outcomes.

Religious variance in willingness to donate may reflect a complex interplay of local beliefs, familial influences, religious interpretations, and prior exposure to donation-related advocacy. While willingness was observed among Hindu and Jain participants in our study, this should not be taken as indicative of inherent religious predispositions, but rather as suggestive of the social and informational environments surrounding the participants. These findings highlight the importance of culturally tailored counseling that respectfully engages with individual belief systems. These observations partially support the conclusions of Gupta et al., who identified religious beliefs as a barrier to organ and tissue donation in India [10]. This underlines the necessity of culturally sensitive approaches when addressing eye donation in diverse communities.

Socioeconomic status also played a critical role in influencing donation willingness. The upper socioeconomic class showed the highest willingness at 30.0%, followed by the lower middle and upper middle classes. No willingness was expressed by participants from the upper lower and lower socioeconomic groups. These findings are consistent with Gupta et al., who noted that eye donation awareness varies widely and is generally higher among individuals with better educational and financial backgrounds [10].

Education emerged as a strong predictor of willingness to donate. While none of the illiterate participants consented to eye donation, 27.8% of graduates expressed willingness, indicating a positive correlation between educational attainment and donation intent. This supports the findings of Arya et al., who identified illiteracy as a strong predictor of ignorance regarding eye donation [14].

Although awareness increased across all demographic categories, this did not always translate into a willingness to donate. This discrepancy suggests that factors beyond knowledge - such as misconceptions, fears, and emotional resistance - might play a more significant role in decision-making. Acharya et al. emphasized that it is often misconceptions about the use of donated tissues, rather than cultural or religious beliefs, that form the primary barriers to donation [11].

Overall, the study emphasizes the crucial role of EDCs in the HCRP. Their ability to develop rapport, provide emotional support, and educate grieving families in a sensitive manner contributed significantly to the improvement in awareness levels. These findings support recommendations by Christy et al., who advocated for training programs at hospitals to promote eye donation through informed and compassionate counseling practices [15].

Despite a significant improvement in awareness post-counseling, the relatively modest increase in actual willingness and conversion suggests that cognitive awareness does not always translate into action. This discrepancy may be influenced by several non-cognitive factors, including acute emotional distress, grief-related denial, decision-making fatigue, and the need for family consensus during the immediate aftermath of death. In such high-stress moments, even well-informed families may defer or avoid donation decisions. Prior studies have also shown that emotional readiness and familial alignment play a critical role in organ donation consent, often outweighing factual knowledge alone. Future interventions should, therefore, consider timing, emotional support, and consensus-building strategies alongside educational efforts to improve donation outcomes.

Limitations

The small sample size in certain subgroups may limit statistical power, so findings should be interpreted with caution. Although reasons for non-willingness weren’t formally assessed, counselors observed concerns like grief, fear of disfigurement, family disagreement, and logistical barriers. Illiteracy may overlap with unmeasured confounders, such as rural background or limited healthcare access. Additionally, the role of treating staff was not analyzed, though their communication may influence family decisions. Future studies should control for these factors and explore them through qualitative methods.

## Conclusions

This study demonstrates that counseling significantly improves awareness about corneal donation, yet translating this awareness into willingness remains challenging, with only a modest 10.3% conversion rate. Factors like education, socioeconomic status, and cultural beliefs influenced willingness to donate. To bridge this gap, strategies such as involving religious leaders, leveraging social influencers, and promoting pre-mortem donor registration are recommended. These measures can ease decision-making during grief and enhance the effectiveness of the HCRP in reducing corneal blindness in India.

## Appendices

Awareness questionnaire on eye donation (13 items)

1. Have you heard about eye or cornea donation before?

2. Do you know that corneas can be donated after death?

3. Do you think eye donation can help restore vision in blind individuals?

4. Are you aware that only the cornea (not the entire eye) is used for transplantation?

5. Do you know that eye donation must occur within 4-6 hours after death?

6. Do you know that eye donation does not lead to facial disfigurement of the deceased?

7. Are you aware that eye donation can be done irrespective of age?

8. Do you know that religious practices generally support eye donation?

9. Are you aware that people with spectacles or cataracts can also donate their eyes?

10. Do you know that there is no cost to the family for donating eyes?

11. Have you heard of any eye bank or eye donation program in your area?

12. Do you know whom to contact in a hospital to initiate the eye donation process?

13. Are you aware that one eye donor can help restore vision in two people?

Each item is intended to be answered as Yes/No/Don't Know, and can be scored 1 for a correct (Yes) answer and 0 for No or Don't Know
